# No association between use of tenofovir disoproxil fumarate, etravirine, or integrase-strand transfer inhibitors and acquisition or severe outcomes of SARS-CoV-2 infection in people with HIV in the Netherlands

**DOI:** 10.1097/QAD.0000000000003577

**Published:** 2023-04-13

**Authors:** Myrthe L. Verburgh, Marc van der Valk, Bart J.A. Rijnders, Peter Reiss, Ferdinand W.N.M. Wit

**Affiliations:** a Amsterdam UMC location University of Amsterdam; b Amsterdam Institute for Infection and Immunity, Infectious Diseases; c Amsterdam Public Health, Global Health; d Amsterdam Institute for Global Health and Development; e HIV Monitoring Foundation, Amsterdam; f Erasmus Medical Center, Departments of Internal Medicine & Medical Microbiology, Rotterdam; g Amsterdam UMC location University of Amsterdam, Global Health, Amsterdam, The Netherlands.

## Abstract

In two Dutch observational cohorts of people with HIV, the use of TDF, ETR, or INSTIs was not independently associated with either the risk of incident SARS-CoV-2 infection or severe COVID-19 outcomes, as was suggested by previous observational and molecular docking studies. Our findings do not support a strategy of modifying antiretroviral therapy to include these agents to protect against SARS-CoV-2 infection and severe COVID-19 outcomes.

Since the start of the COVID-19 pandemic, several studies have tried to determine factors associated with acquisition of and clinical outcome of SARS-CoV-2 infection in people with HIV (PWH). Recent observational studies have suggested a protective effect of tenofovir disoproxil fumarate (TDF) against acquisition of SARS-CoV-2 [1,2] and severe COVID-19 outcomes [1,3,4], whereas other studies found no benefit of TDF or tenofovir alafenamide (TAF) in PWH [5,6] or adults without HIV [7,8].

Etravirine (ETR) and the integrase-strand transfer inhibitors (INSTIs) – specifically raltegravir (RAL) and dolutegravir (DTG) – were proposed as potential inhibitors of two major SARS-CoV-2 proteins in a molecular docking [9] and molecular dynamics simulation study [10]. One recent study showed that in-vitro docking by SARS-CoV-2 to the ACE2 receptor is inhibited by DTG and ETR [11]. Thus far, no studies in PWH have reported epidemiological evidence for a protective effect of the use of INSTIs or ETR against acquiring SARS-CoV-2 infection and severe COVID-19 outcomes.

We investigated the association between the abovementioned antiretrovirals and incident SARS-CoV-2 infection and COVID-19-associated hospitalization and/or death in two Dutch observational cohorts of PWH.

First, we used data from the COVID-19 substudy of the AGE_h_IV cohort collected from September 2020 until April 2021 [12]. PWH and participants without HIV were assessed every 6 months for incident SARS-CoV-2 infection. Incident SARS-CoV-2 infection was defined as positive combined IgA/IgM/IgG SARS-CoV-2 nucleocapsid (N) antibody assay or a self-reported positive PCR test in participants without detectable N-antibodies. We previously reported that younger age and sub-Saharan African origin, but not HIV-status, were independently associated with higher risk of incident SARS-CoV-2 infection. However, we did not investigate the association with specific antiretrovirals in PWH.

Second, we used data from the Dutch national observational HIV cohort (ATHENA), containing data of more than 95% of PWH in care in one of the 24 HIV-treatment centers in the Netherlands [13]. Within this cohort, we recently reported that the risk of severe COVID-19 outcomes was increased in individuals with uncontrolled HIV replication, low CD4^+^ cell count and prior AIDS, independently of general risk factors such as age, comorbidity burden, and non-Western origin (F.W.N.M. Wit, P. Reiss, B. Rijnders, M. van der Valk, in preparation), but potential associations with specific antiretrovirals were not extensively analyzed.

Extending our earlier analyses, we now assessed whether use of TDF, ETR, and INSTIs were associated with the risk of incident SARS-CoV-2 infection in the AGE_h_IV COVID-19 substudy and the risk of COVID-19-associated hospitalization and/or death in the ATHENA cohort. Associations between the aforementioned antiretrovirals and outcomes were assessed using multivariable logistic regression, both unadjusted and adjusted for age, sex at birth, ethnic origin, total comorbidity count (cardiovascular disease; non-AIDS-defining cancer; chronic kidney disease; diabetes mellitus; hypertension; and/or obesity (BMI ≥30 kg/m^2^), prior AIDS, current CD4^+^ cell count (categorized <200, 200–499, ≥500 cells/μl), and current HIV-1 viral load (categorized ≤200 or >200 copies/ml).

In the current analysis, 239 PWH using antiretroviral therapy (ART) from the AGE_h_IV COVID-19 substudy were included. Median age was 62.0 years [interquartile range (IQR) 57.7–67.2], 92.1% were men, and participants were living with HIV for a median of 21.6 years (IQR 15.2–27.2). All participants, except one (99.6%), were virally suppressed (<200 copies/ml) with a current CD4^+^ cell count of 670 cells/μl (IQR 530–834). By April 2021, 29 of 239 PWH had an incident SARS-CoV-2 infection. Those with an incident infection were significantly younger and more often of sub-Saharan African origin compared with those without incident SARS-CoV-2 infection (data not shown). ART regimen did not differ significantly between both groups.

For the ATHENA cohort, 2189 PWH using ART while being diagnosed with COVID-19 were included. Median age was 50.1 years (IQR 40.5–57.7), 80.3% were men, and participants were living with HIV for a median of 12.5 years (IQR 7.2–18.3). About 98.3% had an HIV-1 viral load less than 200 copies/ml and current CD4^+^ cell count of 710 cells/μl (IQR 530–900). One hundred fifty-eight participants had severe COVID-19: hospitalization (*n* = 149) and/or death (*n* = 29; 9/29 deaths occurred in individuals who were not hospitalized). Compared with those with mild COVID-19, participants with severe COVID-19 were significantly older, more often of non-white origin, had more comorbidities, and were living with HIV for more years. Moreover, those with severe COVID-19 had lower median nadir CD4^+^ cell count [153 (IQR 60–262) vs. 260 (130–390) cells/μl, *P* < 0.001] and lower median current CD4^+^ cell count [593 (IQR 403–830) vs. 720 (IQR 540–910) cells/μl, *P* < 0.001]. Those with severe COVID-19 outcomes were more often on a protease inhibitor-containing ART-regimen (22.2 vs. 12.9%), but otherwise ART-regimen did not differ significantly between the groups.

Use of TDF, ETR, or INSTIs was not significantly associated with risk of incident SARS-CoV-2 infection, or severe COVID-19 (Table 1) in both unadjusted and adjusted analyses. Moreover, no associations between use of other antiretrovirals and incident SARS-CoV-2 infection or severe COVID-19 were observed.

**Table 1:** Association between antiretroviral agents and incident SARS-CoV-2 infection in 239 AGE_h_IV COVID-19 substudy participants with known antiretroviral therapy regimen and association between antiretrovirals and severe COVID-19 (hospitalization and/or death) in 2189 ATHENA cohort participants.

	Association between antiretrovirals and incident SARS-CoV-2 infection (AGE_h_IV COVID-19 substudy)					Association between antiretrovirals and severe COVID-19 (hospitalization and/or death) (ATHENA cohort)				
		Unadjusted		Adjusted^a^			Unadjusted		Adjusted^a^	
	*n*/*N*^b^	OR (95% CI)	*P*	OR (95% CI)	*P*	*n*/*N*^b^	OR (95% CI)	*P*	OR (95% CI)	*P*
NRTI backbone										
- Lamivudine only	0/7	–	–	–	–	8/157	0.83 (0.38–1.81)	0.63	0.50 (0.21–1.20)	0.12
- Abacavir-based	2/28	0.65 (0.13–3.28)	0.61	0.56 (0.11–2.95)	0.50	35/339	1.77 (1.10–2.86)	0.018	1.39 (0.83–2.35)	0.21
- Tenofovir alafenamide-based	17/107	1.61 (0.65–3.94)	0.30	1.33 (0.53–3.38)	0.55	67/946	1.18 (0.78–1.77)	0.43	1.01 (0.65–1.56)	0.96
- Tenofovir disoproxil fumarate-based	8/76	REF	REF	REF	REF	39/640	REF	REF	REF	REF
- No NRTI	2/21	0.89 (0.18–4.57)	0.89	0.96 (0.18–5.17)	0.96	9/107	1.42 (0.67–3.01)	.37	0.68 (0.29–1.58)	0.37
INSTI										
- Bictegravir	6/30	2.00 (0.55–7.27)	0.29	2.15 (0.56–8.26)	0.26	21/282	0.93 (0.55–1.59)	0.80	0.89 (0.50–1.60)	0.70
- Dolutegravir	5/45	REF	REF	REF	REF	50/626	REF	REF	REF	REF
- Elvitegravir/cobicistat	6/41	1.37 (0.38–4.89)	.63	0.77 (0.19–3.14)	.72	15/349	0.52 (0.29–0.94)	.029	0.80 (0.43–1.50)	0.50
- Raltegravir	1/12	0.73 (0.08–6.89)	0.78	0.97 (0.10–9.65)	0.98	7/51	1.83 (0.79–4.28)	.16	1.54 (0.61–3.90)	0.37
- No INSTI	11/111	0.88 (0.29–2.69)	0.82	0.83 (0.26–2.64)	0.75	65/881	0.92 (0.63–1.35)	0.66	0.88 (0.58–1.34)	0.54
NNRTI										
- Doravirine	2/11	2.00 (0.24–16.61)	0.52	2.59 (0.27–25.02)	0.41	6/140	0.49 (0.18–1.33)	0.16	0.60 (0.20–1.79)	0.36
- Efavirenz	2/20	REF	REF	REF	REF	12/142	REF	REF	REF	REF
- Etravirine	0/1	–	–	–	–	1/11	1.08 (0.13–9.20)	0.94	0.67 (0.07–6.62)	0.72
- Nevirapine	4/49	0.80 (0.13–4.76)	0.81	0.74 (0.11–5.02)	0.76	18/227	0.93 (0.44–2.00)	0.86	0.86 (0.38–1.99)	0.73
- Rilpivirine	2/13	1.64 (0.20–13.34)	0.65	2.22 (0.24–20.23)	0.48	11/172	0.74 (0.32–1.73)	0.49	1.07 (0.43–2.65)	0.89
- No NNRTI	19/145	1.36 (0.29–6.32)	0.70	1.15 (0.22–6.03)	0.87	110/1497	0.86 (0.46–1.60)	0.64	1.02 (0.51–2.01)	0.97
PI										
- Atazanavir/booster	1/5	5.25 (0.39–71.42)	0.21	9.72 (0.61–155.06)	0.11	2/22	0.72 (0.16–3.14)	0.67	0.50 (0.09–2.74)	0.42
- Darunavir/booster	2/44	REF	REF	REF	REF	33/270	REF	REF	REF	REF
- Lopinavir/booster	0/2	–	–	–	–	0/5	–	–	–	–
- No PI	26/188	3.37 (0.77–14.77)	0.11	3.51 (0.78–15.92)	.10	123/1892	0.50 (0.33–0.75)	<0.001	0.73 (0.47–1.15)	0.17

Values represent odds ratios (OR) with 95% confidence interval using logistic regression.

a Adjusted for age, sex at birth, ethnic origin, total comorbidities count, prior AIDS, current CD4^+^ cell count, and current HIV-1 viral load.

b Number of participants with severe COVID-19 (hospitalization and/or death) per total number of participants for each variable category. CI, confidence interval; INSTI, integrase strand transfer inhibitors; NNRTI, nonnucleoside reverse transcriptase inhibitors; NRTI, nucleoside reverse transcriptase inhibitors; OR, odds ratio; *P*, *P*-value; PI, protease inhibitors; REF, reference group.

Our analyses in two cohorts of PWH support previous findings that TDF does not protect against SARS-CoV-2 infection or severe COVID-19 outcomes [5,6]. Similar to our analyses, these studies adjusted their outcome for baseline participant characteristics, such as country of origin, socioeconomic status, current CD4^+^ cell count and CD4/CD8 ratio, years on ART, presence of diabetes, chronic kidney disease, and metabolic disease [5]. Importantly, studies suggesting a protective effect of TDF in PWH did not adjust for these participant characteristics [2–4]. It is likely that the presence of risk factors such as higher age and comorbidities influence the choice of ART regimen, which may confound the association between ART use and COVID-19 outcomes, thereby explaining the difference in study findings.

With regard to INSTIs, two clinical studies likewise found no protective effect of INSTIs against acquisition of SARS-CoV-2 infection in PWH [2,6].

In conclusion, in two Dutch observational cohorts of PWH, the use of TDF, ETR, or INSTIs was not independently associated with a reduced risk of incident SARS-CoV-2 infection or severe COVID-19 outcomes. Our findings do not support a strategy of modifying ART to include these antiretrovirals to protect against SARS-CoV-2 infection and severe COVID-19.

## Acknowledgements

AGE_h_IV Cohort Study Group: Scientific oversight and coordination: P. Reiss (principal investigator), F.W.N.M. Wit, M. van der Valk, A. Boyd, M.L. Verburgh, I.A.J. van der Wulp, M.C. Vanbellinghen, C.J. van Eeden (Amsterdam University Medical Centers (Amsterdam UMC), University of Amsterdam, Department of Global Health and Amsterdam Institute for Global Health and Development (AIGHD).

M.F. Schim van der Loeff (co-principal investigator), J.C.D. Koole, L. del Grande, I. Agard (Public Health Service of Amsterdam, Department of Infectious Diseases).

Data management: S. Zaheri, M.M.J. Hillebregt, Y.M.C. Ruijs, D.P. Benschop, A. el Berkaoui (HIV Monitoring Foundation).

Statistical support: A. Boyd, F.W.N.M. Wit (HIV Monitoring Foundation).

Central laboratory support: N.A. Kootstra, A.M. Harskamp-Holwerda, I. Maurer, M.M. Mangas Ruiz, B.D.N. Boeser-Nunnink, O.S. Starozhitskaya (Amsterdam UMC, Laboratory for Viral Immune Pathogenesis and Department of Experimental Immunology).

L. van der Hoek, M. Bakker, M.J. van Gils (Amsterdam UMC, Department of Medical Microbiology and Infection Prevention, Laboratory of Experimental Virology).

Project management and administrative support: L. Dol, G. Rongen (AIGHD).

Participating HIV physicians and nurses: S.E. Geerlings, A. Goorhuis, J.W.R. Hovius, F.J.B. Nellen, J.M. Prins, T. van der Poll, M. van der Valk, W.J. Wiersinga, M. van Vugt, G. de Bree, B. A. Lemkes, V. Spoorenberg, F.W.N.M. Wit; J. van Eden, F.J.J. Pijnappel, A. Weijsenfeld, S. Smalhout, I.J. Hylkema - van den Bout, C. Bruins, M.E. Spelbrink (Amsterdam UMC, Division of Infectious Diseases).

Other collaborators: P.G. Postema (Amsterdam UMC, Department of Cardiology); P.H.L.T. Bisschop (Amsterdam UMC, Division of Endocrinology and Metabolism); E. Dekker (Amsterdam UMC, Department of Gastroenterology); N. van der Velde, R. Franssen (Amsterdam UMC, Division of Geriatric Medicine); J.M.R. Willemsen, L. Vogt (Amsterdam UMC, Division of Nephrology); P. Portegies, G.J. Geurtsen (Amsterdam UMC, Department of Neurology); I. Visser, A. Schadé (Amsterdam UMC, Department of Psychiatry); P.T. Nieuwkerk (Amsterdam UMC, Department of Medical Psychology); R.P. van Steenwijk, R.E. Jonkers (Amsterdam UMC, Department of Pulmonary medicine); C.B.L.M. Majoie, M.W.A. Caan (Amsterdam UMC, Department of Radiology); B.J.H. van den Born, E.S.G. Stroes (Amsterdam UMC, Division of Vascular Medicine); S. van Oorspronk (HIV Vereniging Nederland).

The ATHENA observational cohort study

Clinical centers (*∗ denotes site coordinating physician*): Amsterdam UMC, AMC site, Amsterdam: HIV treating physicians: M. van der Valk∗, S.E. Geerlings, A. Goorhuis, V.C. Harris, J.W. Hovius, B. Lempkes, F.J.B. Nellen, T. van der Poll, J.M. Prins, P. Reiss, V. Spoorenberg, M. van Vugt, W.J. Wiersinga, F.W.M.N. Wit. HIV nurse consultants: C. Bruins, M. van Duinen, J. van Eden, A.M.H. van Hes, F.J.J. Pijnappel, S.Y. Smalhout, A.M. Weijsenfeld. HIV clinical virologists/ chemists: N.K.T. Back, B. Berkhout, M.T.E. Cornelissen, R. van Houdt, M. Jonges, S. Jurriaans, C.J. Schinkel, K.C. Wolthers, H.L. Zaaijer. Amsterdam UMC, VUmc site, Amsterdam: HIV treating physicians: E.J.G. Peters∗, M.A. van Agtmael, R.S. Autar, M. Bomers, K.C.E. Sigaloff. HIV nurse consultants: M. Heitmuller, L.M. Laan, J. Steiner. HIV clinical virologists/chemists: N.K.T. Back, B. Berkhout, M.T.E. Cornelissen, R. van Houdt, M. Jonges, S. Jurriaans, C.J. Schinkel, K.C. Wolthers, H.L. Zaaijer. Emma Kinderziekenhuis (Amsterdam UMC, AMC site), Amsterdam: HIV treating physicians: M. van der Kuip, D. Pajkrt. HIV nurse consultants: A.M. Weijsenfeld. Admiraal De Ruyter Ziekenhuis, Goes: HIV treating physicians: M. van den Berge∗, A. Stegeman. HIV nurse consultants: S. Baas, L. Hage de Looff. HIV clinical virologists/chemists: P. van Keulen, J. Stohr, B. Wintermans. Catharina Ziekenhuis, Eindhoven: HIV treating physicians: M.J.H. Pronk∗, H.S.M. Ammerlaan. HIV nurse consultants: E.S. de Munnik. HIV clinical virologists/chemists: B. Deiman, A.R. Jansz, V. Scharnhorst, J. Tjhie, M.C.A. Wegdam. DC Klinieken Lairesse – Hiv Focus Centrum, Amsterdam: HIV treating physicians: M. van der Valk∗, A. van Eeden, E. Hoornenborg, J. Nellen. HIV nurse consultants: L.J.M. Elsenburg, H. Nobel. HIV clinical virologists/chemists: C.J. Schinkel. ETZ (Elisabeth-TweeSteden Ziekenhuis), Tilburg: HIV treating physicians: M.E.E. van Kasteren∗, M.A.H. Berrevoets, A.E. Brouwer. HIV nurse specialist: B.A.F.M. de Kruijf-van de Wiel. HIV nurse consultants: A. Adams, A. Dievelaar-Oomen, R. van Erve, S. Phaf, B. van de Ven. HIV data collection: B.A.F.M. de Kruijf-van de Wiel. HIV clinical virologists/chemists: A.G.M. Buiting, J.L. Murck. Erasmus MC, Rotterdam: HIV treating physicians: C. Rokx∗, A.A. Anas, H.I. Bax, E.C.M. van Gorp, M. de Mendonça Melo, E. van Nood, J.L. Nouwen, B.J.A. Rijnders, C.A.M. Schurink, L. Slobbe, T.E.M.S. de Vries-Sluijs. HIV nurse consultants: N. Bassant, J.E.A. van Beek, M. Vriesde, L.M. van Zonneveld. HIV data collection: J. de Groot. HIV clinical virologists/chemists: J.J.A. van Kampen, M.P.G Koopmans. Erasmus MC Sophia Kinderziekenhuis, Rotterdam: HIV treating physicians: P.L.A. Fraaij, A.M.C. van Rossum, C.L. Vermont. HIV nurse consultants: L.C. van der Knaap.

Flevoziekenhuis, Almere: HIV treating physicians: J. Branger∗, R.A. Douma. HIV nurse consultant: A.S. Cents-Bosma, C.J.H.M. Duijf-van de Ven. HagaZiekenhuis, Den Haag: HIV treating physicians: E.F. Schippers∗, C. van Nieuwkoop, L. Smit. HIV nurse consultants: J. Geilings, S. van Winden. HIV data collection: G. van der Hut. HIV clinical virologists/chemists: N.D. van Burgel. HMC (Haaglanden Medisch Centrum), Den Haag: HIV treating physicians: E.M.S. Leyten∗, L.B.S. Gelinck, F. Mollema. HIV nurse consultants: S. Davids-Veldhuis, C. Tearno, G.S. Wildenbeest. HIV clinical virologists/chemists: E. Heikens. Isala, Zwolle: HIV treating physicians: P.H.P. Groeneveld∗, J.W. Bouwhuis, A.J.J. Lammers. HIV nurse consultants: A.G.W. van Hulzen, S. Kraan, M.S.M. Kruiper. HIV data collection: G.L. van der Bliek, P.C.J. Bor. HIV clinical virologists/chemists: S.B. Debast, G.H.J. Wagenvoort. Leids Universitair Medisch Centrum, Leiden: HIV treating physicians: A.H.E. Roukens∗, M.G.J. de Boer, H. Jolink, M.M.C. Lambregts, A.H.E. Roukens, H. Scheper. HIV nurse consultants: W. Dorama, N. van Holten. HIV clinical virologists/chemists: E.C.J. Claas, E. Wessels. Maasstad Ziekenhuis, Rotterdam: HIV treating physicians: J.G. den Hollander∗, R. El Moussaoui, K. Pogany. HIV nurse consultants: C.J. Brouwer, D. Heida-Peters, E. Mulder, J.V. Smit, D. Struik-Kalkman.

HIV data collection: T. van Niekerk. HIV clinical virologists/chemists: O. Pontesilli, C. van Tienen. Maastricht UMC+, Maastricht: HIV treating physicians: S.H. Lowe∗, A.M.L. Oude Lashof, D. Posthouwer, M.E. van Wolfswinkel. HIV nurse consultants: R.P. Ackens, K. Burgers, J. Schippers. HIV data collection: B. Weijenberg-Maes. HIV clinical virologists/chemists: T.R.A. Havenith, M. van Loo. Medisch Centrum Leeuwarden, Leeuwarden: HIV treating physicians: M.G.A. van Vonderen∗, L.M. Kampschreur. HIV nurse consultants: M.C. van Broekhuizen, S, Faber. HIV clinical virologists/chemists: A. Al Moujahid. Medisch Spectrum Twente, Enschede: HIV treating physicians: G.J. Kootstra∗, C.E. Delsing. HIV nurse consultants: M. van der Burg-van de Plas, L. Scheiberlich. Noordwest Ziekenhuisgroep, Alkmaar: HIV treating physicians: W. Kortmann∗, G. van Twillert∗, R. Renckens, J. Wagenaar. HIV nurse consultants & HIV data collection: D. Ruiter-Pronk, F.A. van Truijen-Oud. HIV clinical virologists/chemists: J.W.T. Cohen Stuart, M. Hoogewerf, W. Rozemeijer, J.C. Sinnige. OLVG, Amsterdam: HIV treating physicians: K. Brinkman∗, G.E.L. van den Berk, K.D. Lettinga, M. de Regt, W.E.M. Schouten, J.E. Stalenhoef, J. Veenstra, S.M.E. Vrouenraets. HIV nurse consultants: H. Blaauw, G.F. Geerders, M.J. Kleene, M. Knapen, M. Kok, I.B. van der Meché, A.J.M. Toonen, S. Wijnands, E. Wttewaal. HIV clinical virologists: D. Kwa, T.J.W. van de Laar. Radboudumc, Nijmegen: HIV treating physicians: R. van Crevel∗, K. van Aerde, A.S.M. Dofferhoff, S.S.V. Henriet, H.J.M. ter Hofstede, J. Hoogerwerf, O. Richel. HIV nurse consultants: M. Albers, K.J.T. Grintjes-Huisman, M. de Haan, M. Marneef. HIV clinical virologists/chemists: F.F. Stelma. HIV clinical pharmacology consultant: D. Burger. Rijnstate, Arnhem: HIV treating physicians: E.H. Gisolf∗, M. Claassen, R.J. Hassing, HIV nurse consultants: G. ter Beest, P.H.M. van Bentum, M. Gelling, N. Langebeek. HIV clinical virologists/chemists: C.M.A. Swanink, M. Klein Velderman. Spaarne Gasthuis, Haarlem: HIV treating physicians: S.F.L. van Lelyveld∗, R. Soetekouw. HIV nurse consultants: L.M.M. van der Prijt, J. van der Swaluw. HIV clinical virologists/chemists: J.S. Kalpoe, A. Vahidnia, A. Wagemakers. Medisch Centrum Jan van Goyen, Amsterdam: HIV treating physicians: F.N. Lauw, D.W.M. Verhagen. HIV nurse consultants: M. van Wijk. Universitair Medisch Centrum Groningen, Groningen: HIV treating physicians: W.F.W. Bierman∗, M. Bakker, R.A. van Bentum, M.A. van den Boomgaard, J. Kleinnijenhuis, E. Kloeze, A. Middel, D.F. Postma, Y. Stienstra, M. Wouthuyzen-Bakker. HIV nurse consultants: A. Boonstra, H. de Groot-de Jonge, M.M.M. Maerman, P.A. van der Meulen, D.A. de Weerd. HIV clinical virologists/chemists: K.J. van Eije, M. Knoester, C.C. van Leer-Buter, H.G.M. Niesters. Beatrix Kinderziekenhuis (Universitair Medisch Centrum Groningen), Groningen: HIV treating physicians: E.H. Schölvinck, A.R. Verhage. HIV nurse consultants: H. de Groot-de Jonge. HIV clinical virologists/chemists: M. Knoester, C.C. van Leer-Buter, H.G.M. Niesters. Universitair Medisch Centrum Utrecht, Utrecht: HIV treating physicians: T.Mudrikova∗, R.E. Barth, A.H.W. Bruns, P.M. Ellerbroek, M.P.M. Hensgens, J.J. Oosterheert, E.M. Schadd, A. Verbon, B.J. van Welzen. HIV nurse consultants: K. Aarsman, B.M.G. Griffioen-van Santen, I. de Kroon. HIV data collection: M. van Berkel, C.S.A.M. van Rooijen. HIV clinical virologists/chemists: L.M. Hofstra, R. Schuurman, A.M.J. Wensing. Wilhelmina Kinderziekenhuis, UMC Utrecht, Utrecht: HIV treating physicians: S.P.M. Geelen, Y.G.T. Loeffen, T.F.W. Wolfs. HIV nurse consultants: N. Nauta. Curaçao Medical Center, Willemstad (Curaçao): HIV treating physicians: E.O.W. Rooijakkers, D. van de Wetering. HIV nurse consultants: A. Alberto. Data collection: I. der Meer.

Coordinating center: Board of directors: M. van der Valk, S. Zaheri. HIV data analysis: A.C. Boyd, D.O. Bezemer, A.I. van Sighem, C. Smit, F.W.M.N. Wit. Data HIV data management and quality control: M.M.J. Hillebregt, T.J. Woudstra, T. Rutkens. HIV data monitoring: D. Bergsma, N.M. Brétin, K.J. Lelivelt, L. van de Sande, K.M. Visser

S.T. van der Vliet. HIV data collection: F. Paling, L.G.M. de Groot-Berndsen, M. van den Akker, R. Alexander, Y. Bakker, A. El Berkaoui, M. Bezemer-Goedhart, E.A. Djoechro, M. Groters, L.E. Koster, C.R.E. Lodewijk, R.J. Loenen, E.G.A. Lucas, S. van Meerveld, L. Munjishvili, B.M. Peeck, C.M.J. Ree, R. Regtop, A.F. van Rijk, Y.M.C. Ruijs-Tiggelman, P.P. Schnörr, M.J.C. Schoorl, E.M Tuijn, D.P. Veenenberg, E.C.M Witte. Patient registration: Y.M.C. Ruijs-Tiggelman, D. Bergsma.

### Conflicts of interest

M. van der Valk through his institution has received independent scientific grant support and consultancy fees from Gilead Sciences, MSD, and ViiV Healthcare, for which honoraria were all paid to his institution. B.R. has received research grants from Gilead, research grants from Merck Sharp and Dohme, and honoraria from Jansen-Cilag, BMS, Pfizer, and ViiV. P.R. through his institution has received independent scientific grant support from Gilead Sciences, Janssen Pharmaceuticals Inc, Merck & Co, and ViiV Healthcare, and has served on scientific advisory boards for Gilead Sciences, ViiV Healthcare, and Merck & Co honoraria for which were all paid to his institution. F.W.N.M.W. has served on scientific advisory boards for ViiV Healthcare and Gilead sciences. M.L.V. declares no competing interests.

The AGE_h_IV COVID-19 substudy was supported in part through an investigator-initiated study grant from ViiV Healthcare. The parent AGE_h_IV Cohort Study was supported by The Netherlands Organization for Health Research and Development [ZonMW, grant number 30002000] and AIDS Fonds [grant number 2009063], and in part by unrestricted research grants from Gilead Sciences; ViiV Healthcare; Janssen Pharmaceuticals N.V.; and Merck Sharp & Dohme Corp. The ATHENA cohort is managed by the HIV Monitoring Foundation (*Stichting hiv monitoring*) and supported by a grant from the Dutch Ministry of Health, Welfare and Sport through the Center for Infectious Disease Control of the National Institute for Public Health and the Environment.

Informed consent was obtained from all participants. The AGE_h_IV cohort study was approved by the ethics committee of the Amsterdam UMC, location AMC, and is registered at www.clinicaltrials.gov (NCT01466582). The ATHENA cohort was approved by the institutional review boards of all participating HIV treatment centers.
